# Metastasis of an occult pulmonary carcinoma into meningioma: a case report

**DOI:** 10.1186/s12957-015-0714-3

**Published:** 2015-10-05

**Authors:** Janez Ravnik, Maja Ravnik, Gorazd Bunc, Ivana Glumbic, Erzebet Tobi-Veres, Tomaz Velnar

**Affiliations:** Department of Neurosurgery, University Medical Centre Maribor, Ljubljanska 5, 2000 Maribor, Slovenia; Department of Oncology, University Medical Centre Maribor, Ljubljanska 5, 2000 Maribor, Slovenia; Department of Pathology, University Medical Centre Maribor, Ljubljanska 5, 2000 Maribor, Slovenia; Department of Neurology, General Hospital Murska Sobota, 9000 Murska Sobota, Slovenia

**Keywords:** Tumour-to-tumour metastasis, Meningioma, Carcinoma, Surgery, Brain

## Abstract

Tumour-to-tumour metastasis is an infrequent pathological phenomenon. Meningioma is the most common intracranial tumour where metastatic deposits may be found, the majority of which arise from breast and lung cancers. We describe an unusual case of occult pulmonary carcinoma metastasis into the intracranial meningioma. A 77-year old lady presented with acutely deteriorating hemiparesis. Her previous medical history was unremarkable. Radiological imaging revealed an expansive lesion, classified as meningioma, which was located parasagittally in the right premotor area. A well-capsulated tumour attached to the dura was removed surgically. The pathological examination demonstrated a mixture of angiomatous meningioma and pulmonary adenocarcinoma. Possible explanations for the development of a composite tumour and pathophysiology are described.

## Background

Tumours of non-neural origin, besides being aggressive locally, metastasize frequently to the central nervous system, and this represents a relatively common complication of most cancers from elsewhere in the body, including carcinoma of the lung and breast and malignant melanoma [1–4]. On the other hand, primary brain tumours that are also characterised by infiltrative growth into the surrounding brain tissue are principally confined to the central nervous system, and metastases of these tumours to other locations are exceptional [4–6].

In addition to the rising incidence of primary and secondary brain tumours, tumour-to-tumour metastasis may also be encountered in clinical practice [1, 3]. Although these metastases from one tumour into another, which is also called tumour-to-tumour phenomenon, are a very rare pathological entity, they represent a well-recognised phenomenon [2, 7, 8]. Meningiomas have been described as the most frequent intracranial tumours to host metastases, with cancers of breast and lung being the most frequent primary sites [1, 3, 7, 9–12]. Other malignancies have been only exceptionally found to metastasise into meningioma, for example renal, genitourinary, gastrointestinal, prostate and parotid tumours and lymphoma [1, 7, 11].

Clinically, a variety of signs and symptoms may occur [5]. Epileptic seizures of different types, which may be present in up to 85 % of patients, are one of the most common symptoms. Others include headaches, nausea and dizziness, sudden or insidious cognitive and mood deteriorations, as well as sensory and motor disturbances in terms of localised or generalised limb weakness and cranial nerve dysfunction due to affection of eloquent brain zones or cranial nerves itself [1, 5, 6]. The differential diagnosis of such lesions may include a primary cerebral malignancy or a metastatic tumour [1, 3]. Because metastases develop into meningiomas and may simulate the metastatic disease both clinically and pathohistologically, they present a unique differential diagnostic dilemma [9].

Surgical excision is the principal form of treatment of patients with a history of extracranial cancer although stereotactic radiosurgery is becoming a desirable alternative therapeutic option, especially in eloquent areas of the brain [13–15]. A concomitant solitary intracranial mass must be preceded by radiographical determination of the extent of the primary lesion and possible metastatic deposits [13]. In order to diagnose these tumours, various imaging modalities may be employed, including computer tomography (CT) and magnetic resonance imaging (MRI) [8].

An unusual case of adenocarcinoma of the lung metastasis into the intracranial meningioma in a 77-year old lady is presented.

## Case presentation

A 77-year old lady with a known expansive lesion of the right frontal lobe was admitted to the department due to a rapid deterioration of left-sided hemiparesis. She has been followed up constantly at the neurosurgical outpatient clinic as a result of an expansive lesion, radiologically characterised as meningioma. The lesion has been known for many years, and according to radiological imaging, it had been persistingly unchanged. Until now, the woman was in a good medical condition and did not report of any difficulties in relation to this lesion. However, her neurological condition deteriorated suddenly with weakness of the left limbs. At home, she was walking with progressive difficulty, the gait was unstable and in a few hours, she was not able to stand and walk any more. Additionally, she complained of frontal headache and nausea. Neither loss of consciousness nor seizures were reported by the relatives.

During a neurological examination, no consciousness or cognitive deficits were found. Testing of cranial nerves was normal. There was marked muscle weakness of the left side, equally pronounced in the upper and lower extremity. On the left, the muscle tone was slightly lowered. In comparison with the right side, the reflexes in the left upper and lower extremities were weaker. Walking was not possible.

**Fig. 1:**
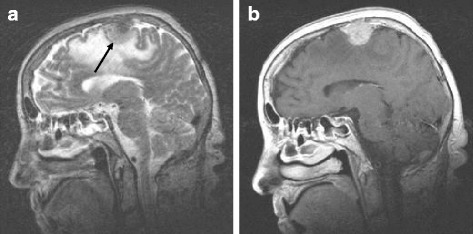
A sagittal T2-weighted spin echo image showing a cortical meningioma in the motor area (*arrow*) surrounded by extensive oedema (**a**). On T1-weighted spin echo image with contrast application (**b**), the tumour enhanced homogenously

MRI of the head revealed progression of the tumour, measuring 3 cm in diameter. It was located parasagittally in the right premotor area, surrounded with an extensive oedema, compressing the cortex. After contrast application, it was enhanced homogenously (Fig. 1). Following antioedematous therapy with intravenous injections of dexamethasone, the condition briefly improved but worsened again despite further aggressive medicamentous treatment. Surgery was indicated. A right parietal parasagittal craniotomy was performed, nicely exposing the oedematous brain tissue of premotor cortex with the tumour, which was removed completely.

**Fig. 2:**
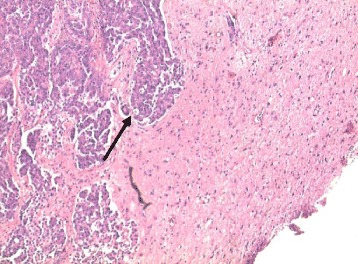
Histological sample of angiomatous meningioma with cords and sheets of malignant cells from pulmonary carcinoma infiltrating the meningioma tissue (haematoxylin and eosin staining, original magnification ×200)

**Fig. 3:**
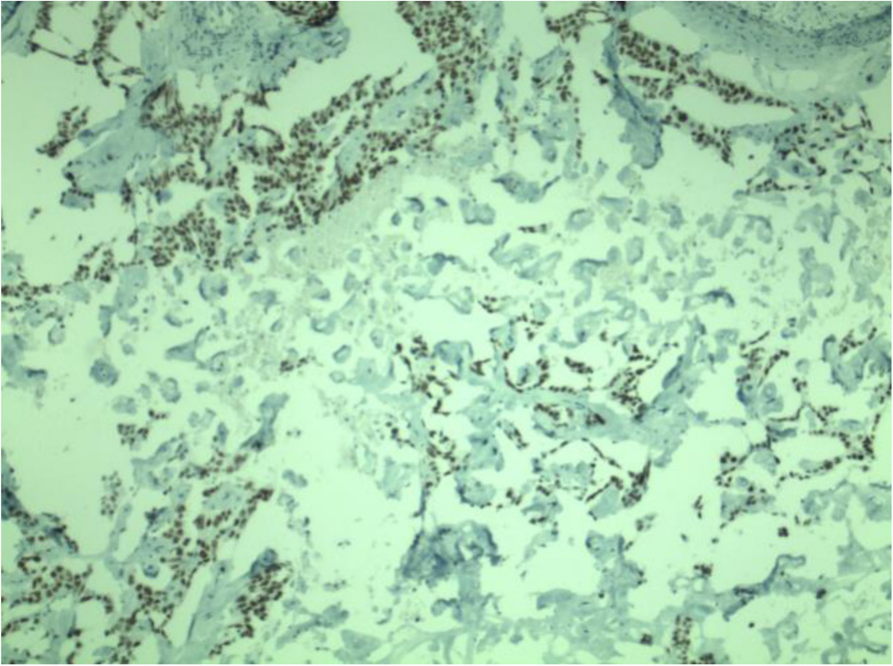
Histological sample with malignant cells showing TTF-1 nuclear immunoreactivity (coloured in *brown*) (TTF-1 stain, original magnification ×40)

Histologically, the tumour was an angiomatous meningioma. In the tumour mass, however, malignant tissue arranged in cords and sheets and glandular formations of atypical epithelial cells were present (Fig. 2). Malignant cells were showing thyroid transcription factor-1 (TTF-1) nuclear immunoreactivity (Fig. 3). TTF-1 is used as a marker to determine the origin of another tumour from lung or thyroid. The frequency of TTF-1 immunoreactivity in extrapulmonary adenocarcinomas, except the thyroid, is lower than 1 %, and the positivity for TTF-1 may be interpreted as a definite evidence that the tumour originates from the lung. Although the neurological condition of the patient improved after the operation, further diagnostics of the thorax revealed a pulmonary lesion located in the left lung. Its histological result was similar to the metastatic intracranial tumour. No pulmonary symptoms were present, and excellent performance state of the patient allowed appropriate oncological therapy.

## Discussion

Tumour-to-tumour metastasis is a rare entity, first described in 1930 by Fried [16]. It denotes that one primary tumour has metastatic foci of another primary tumour in the same individual. This phenomenon is uncommon, with less than 100 cases described in literature from its first report [17]. Any organ system may be affected. Although every benign or malignant tumour may be a recipient, the most common malignant tumour as a recipient is the renal cell carcinoma and the most common benign one is meningioma [1, 18–20]. The donor neoplasm is most frequently breast carcinoma, followed by the lung [1, 18]. Some rare cases of metastasis from other primary tumours have been reported, including renal and rarely prostate or genitourinary cancers [19, 21, 22].

Campbell proposed strict criteria for this diagnosis in 1968, which are as follows: (I) existing more than one primary tumour, (II) the recipient is a true neoplasm, (III) the metastatic neoplasm is true metastasis with evident growth in recipient tumour and (IV) exclusion of the tumours that have metastasized to the lymphatic system, where lymphoreticular malignant tumours already exist [23]. According to these, our patient meets all the demanded criteria.

Tumour-to-tumour metastasis in the central nervous system is even rarer [1]. Since the metastasis to leptomeningeal place is common, these metastatic foci can arise next to the meningioma that may result in collision of these two tumour types. In order to differentiate tumour-to-tumour growth from collision tumours, Pamphlett proposed other basic criteria [8]. For the diagnosis of true tumour-to-meningioma metastasis the following criteria should be considered: (I) the metastatic foci should at least partially be enclosed by a rim of histologically different host tumour tissue and (II) the existing primary tumour should be proven and histologically compatible with metastasis. In our case, the lung cancer metastasis to lung known meningioma fails the first Campell’s and the second Pamphlett’s consideration, although retrospectively it fulfils both [8, 23]. Our patient was followed up regularly for years regarding meningioma, which was not changing in size over the time. A sudden enlargement of the meningioma due to intratumoural bleeding and especially due to surrounding oedema causing the neurological deterioration of the patient with subsequent surgical removal of the meningioma, revealed the not known but existing cancer disease. Histological specimen showed meningothelial meningioma with clear inclusion foci of adenocarcinoma. Immunohistochemistry further revealed that the origin could be the lung, which was later confirmed with further imaging.

Meningiomas have been the most common intracranial neoplasms to harbour metastasis, and the reason for a cancer seeding into meningioma is not precisely known. It is suggested that meningiomas, as generally slow growing and indolent tumours, make a suitable destination for cancer metastasis over a period of time due to the clinical and biological characteristics, such as higher incidence among intracranial neoplasms, hypervascularity, slow growth, low metabolic activity and high collagen and lipid content [7, 19, 21]. All of these characteristics create a favourable, non-competitive environment which favours this metastatic expansion [2, 3, 9]. Molecules involved in the disruption of cellular adhesions and immunological influences may also contribute to this phenomenon [21, 24].

Frequently, tumour-to-tumour metastasis is seen in the course of cancer disease and its treatment. It is very unusual that the complications of the tumour-to-tumour metastasis are the first sign of the cancer disease. Clinically, a variety of signs and symptoms may occur [5]. Epileptic seizures of different types, which may be present in up to 85 % of patients, are one of the most common symptoms. Others include headaches, nausea and dizziness, sudden or insidious cognitive and mood deteriorations, as well as sensory and motor disturbances in terms of localised or generalised limb weakness and cranial nerve dysfunction due to affection of eloquent brain zones or cranial nerves itself [1, 5, 6]. The differential diagnosis of such lesions may include a primary cerebral malignancy or a metastatic tumour [1, 3]. Metastasis into meningiomas may simulate a metastatic disease both clinically and pathohistologically, and therefore, they present a unique differential diagnostic dilemma [9].

Surgical excision is the principal form of treatment of patients with a history of extracranial cancer and concomitant solitary intracranial mass and must be preceded by radiographical determination of the extent of the primary lesion and possible metastatic deposits [13]. In order to diagnose these tumours, various imaging modalities may be employed, including CT and MRI [8]. Limitations of these standard radiological imaging techniques, which cannot reliably identify the presence of metastasis within a meningioma, may be supplemented with physiology-based neuroimaging methods, such as perfusion MRI and MR spectroscopy. These may be more useful in noninvasively differentiating tumour histology [17, 25]. Although MR spectroscopy is gaining popularity, definitive results may be set only according to the pathohistological findings [25, 26].

Alongside micro-neurosurgery, stereotactic radiosurgery is a desirable treatment modality [14, 27, 28]. As a primary treatment, it is the most suitable in cases of deep neurosurgically inaccessible or multiple lesions, where conventional forms of radiotherapy by irradiating the whole head are not appropriate due to the greater risk of irradiation tissue damage [14, 28]. Besides tumours, such as vestibular schwannomas, meningiomas, certain primary brain tumours and brain metastases, examples include also arteriovenous malformations. Radiosurgical treatment of lesions requires a source of high energy rays and a method of applying the radiation in an accurate way, conforming to the target volume thus preserving the surrounding tissue [28–30]. In comparison to micro-neurosurgery, the neurological deficits in patients treated with radiosurgical techniques tend to be smaller, although technical limits and the risk of irradiation damage to the brain do exist [14, 28].

## Conclusions

Tumour-to-tumour metastasis is a rare but well-known event, especially in case of malignant tumours. Due to high blood flow in the meningioma vessels, invasive pulmonary carcinoma cells may easily be transported and may survive in the meningioma tissue, with the tumour microenvironment positively influencing the growth of the implanted tumour cells.

## Consent

Written informed consent was obtained from the patient for publication of this case report and any accompanying images. A copy of the written consent is available for review by the Editor-in-Chief of this journal.

## References

[1] Takei H, Powell SZ (2009). Tumor-to-tumor metastasis to the central nervous system. Neuropathology.

[2] Bhargava P, McGrail KM, Manz HJ, Baidas S (1999). Lung carcinoma presenting as metastasis to intracranial meningioma: case report and review of the literature. Am J Clin Oncol.

[3] Baratelli GM, Ciccaglioni B, Dainese E, Arnaboldi L (2004). Metastasis of breast carcinoma to intracranial meningioma. J Neurosurg Sci.

[4] Pedersen PH, Rucklidge GJ, Mørk SJ, Terzis AJ, Engebraaten O, Lund-Johansen M (1994). Leptomeningeal tissue: a barrier against brain tumor cell invasion. J Natl Cancer Inst.

[5] Stalpers LJ, Dieleman EM, van Westing BR, Postma TJ, van Furth WR (2009). Diagnosis and treatment of brain tumours. Ned Tijdschr Tandheelkd.

[6] Velnar T, Smrdel U, Popovic M, Bunc G (2010). Genetic markers in oligodendroglial tumours. Radiol Oncol.

[7] Benedetto N, Perrini P, Scollato A, Buccoliero AM, Di Lorenzo N (2007). Intracranial meningioma containing metastatic colon carcinoma. Acta Neurochir (Wien).

[8] Pamphlett R (1984). Carcinoma metastasis to meningioma. J Neurol Neurosurg Psychiatry.

[9] Bori R, Kiss CA, Huszka E, Szûcs M, Tusa M, Cserni G (2002). A rare case of tumor-to-tumor metastasis: secondary deposits of pulmonary adenocarcinoma in a secretory meningioma. Magy Onkol.

[10] Conzen M, Sollmann H, Schnabel R (1986). Metastasis of lung carcinoma to intracranial meningioma. Case report and review of literature. Neurochirurgia (Stuttg).

[11] Neroni M, Artico M, Pastore FS, Esposito S, Fraioli B (1999). Diaphragma sellae metastasis from colon carcinoma mimicking a meningioma. A case report. Neurochirurgie.

[12] el Sharouni SY, Berfelo MW, Theunissen PH, Jager JJ, de Jong JM (1993). A unique case of intracranial metastasis from lung carcinoma. Clin Neurol Neurosurg.

[13] Velnar T, Bunc G (2008). Iatrogenic metastasis of a benign meningioma to the periosteum at the site of previous craniotomy: a case report. Wien Klin Wochenschr.

[14] Conti A, Pontoriero A, Midili F, Iatì G, Siragusa C, Tomasello C (2015). CyberKnife multisession stereotactic radiosurgery and hypofractionated stereotactic radiotherapy for perioptic meningiomas: intermediate-term results and radiobiological considerations. Springerplus.

[15] Friehs GM, Park MC, Goldman MA, Zerris VA, Noren G, Sampath P (2007). Stereotactic radiosurgery for functional disorders. Neurosurg Focus.

[16] Fried BM (1930). Metastatic inoculation of a meningioma by cancer cells from a bronchiogenic carcinoma. Am J Pathol.

[17] Moody P, Murtagh K, Piduru S, Brem S, Murtagh R, Rojiani AM (2012). Tumor-to-tumor metastasis: pathology and neuroimaging considerations. Int J Clin Exp Pathol.

[18] Lee HE, Kim DH, Cho YH, Kim K, Chae SW, Sohn JH (2011). Tumor-to-tumor metastasis: hepatocellular carcinoma metastatic to parathyroid adenoma. Pathol Int.

[19] Petraki C, Vaslamatzis M, Argyrakos T, Petraki K, Strataki M, Alexopoulos C (2003). Tumor to tumor metastasis: report of two cases and review of the literature. Int J Surg Pathol.

[20] Schmitt HP (1984). Metastases of malignant neoplasms to intracranial tumours: the “tumour-in-a-tumour” phenomenon. Virchows Arch A Pathol Anat Histopathol.

[21] Lanotte M, Benech F, Panciani PP, Cassoni P, Ducati A (2009). Systemic cancer metastasis in a meningioma: report of two cases and review of the literature. Clin Neurol Neurosurg.

[22] Aghi M, Kiehl TR, Brisman JL (2005). Breast adenocarcinoma metastatic to epidural cervical spine meningioma: case report and review of the literature. J Neurooncol.

[23] Campbell LV, Gilbert E, Chamberlain CR, Watne AL (1968). Metastases of cancer to cancer. Cancer.

[24] Caroli E, Salvati M, Giangaspero F, Ferrante L, Santoro A (2006). Intrameningioma metastasis as first clinical manifestation of occult primary breast carcinoma. Neurosurg Rev.

[25] Jun P, Garcia J, Tihan T, McDermott MW, Cha S (2006). Perfusion MR imaging of an intracranial collision tumor confirmed by image-guided biopsy. AJNR Am J Neuroradiol.

[26] Moller-Hartmann W, Herminghaus S, Krings T, Marquardt G, Lanfermann H, Pilatus U (2002). Clinical application of proton magnetic resonance spectroscopy in the diagnosis of intracranial mass lesions. Neuroradiology.

[27] Vachhrajani S, Fawaz C, Mathieu D, Menard C, Cusimano MD, Gentili F (2008). Complications of gamma knife surgery: an early report from 2 Canadian centers. J Neurosurg.

[28] Vesper J, Bölke B, Wille C, Gerber PA, Matuschek C, Peiper M (2009). Current concepts in stereotactic radiosurgery—a neurosurgical and radiooncological point of view. Eur J Med Res.

[29] Shields CB, Guan YT, Almond PR, Garretson HD, Lindberg RD (1993). Radioneurosurgery using the LINAC scalpel: technique, indications, and literature review. J Ky Med Assoc.

[30] Bunc G, Ravnik J, Ravnik M, Velnar T. Partial skull base tumour resection in combination with radiosurgery: an escape procedure or a reasonable solution of treatment? Wien Klin Wochenschr. 2015. [Epub ahead of print]. 10.1007/s00508-015-0787-6 25925166

